# Understanding the complexity of surgical procedures in RCTs: a pilot study to test the application of the MRC framework for evaluating complex healthcare interventions in the operating theatre

**DOI:** 10.1186/1745-6215-12-S1-A148

**Published:** 2011-12-13

**Authors:** Mark O Eveleigh, Natalie S Blencowe, Nicola Mills, Jane M Blazeby

**Affiliations:** 1 Surgical Research Unit, School of Social and Community Medicine, University of Bristol, Bristol, UK

## Background

Several methodological challenges make randomised trials (RCTs) in surgery difficult, and whether surgical interventions are themselves complex is an issue that requires further exploration [1]. Surgical interventions have multiple concomitant parts that may independently and inter-dependently influence outcomes such as the operation itself, surgeon expertise and contextual factors such as team working and elements of pre- and post-operative care. Trials that have failed to account for this complexity may be criticised and results not accepted or implemented. The MRC framework for developing and evaluating complex healthcare interventions highlights the need to use qualitative methods to define and identify individual active components of interventions during early stages of trial design [2]. This study piloted application of the MRC framework in the development of a surgical intervention within an ongoing RCT.

## Materials and methods

Qualitative research methods were applied to two operations within the context of an on-going RCT of pre-operative chemotherapy and surgery for oesophageal cancer (OEO5 trial). Non-participant observation in the operating theatre and video-recording of the surgery itself were undertaken. Digital video recordings were collected directly from equipment routinely used in laparoscopic surgery onto an encrypted hard drive, transferred to a secure server and analysed. Clinical and non-clinical interactions in the operating theatre were recorded.

## Results

The logistics of video recording surgical procedures were explored and operations successfully recorded and transferred to the secure server. Analyses of procedures proved complex, therefore additional software was identified to allow systematic coding of technical parts of the operation. Non-participant observations were divided into issues relating to the intervention itself, its components (patient, surgeon, assistant, anaesthetist, nurses) as well as contextual factors. Specific comments made by any team members were also documented. This enabled generation of a thematic framework for future analyses and allowed triangulation of findings.

## Conclusions

Using qualitative research methods to understand the component parts of surgical interventions is a novel concept but this early work shows it is feasible. Future research will extend to new RCTs in surgery and include case studies of the interventions and how they are delivered (with in-depth interviews with patients, surgeons and other team members as well as video and audio recordings and non-participant observation).This will improve understanding of the complexity of surgical interventions and generate methods to manualise interventions and monitor fidelity to the protocol, meaning that trial results may be more likely to be believed and accurately implemented in practice.
